# Outcomes of Visual Self-Expression in Virtual Reality on Psychosocial Well-Being With the Inclusion of a Fragrance Stimulus: A Pilot Mixed-Methods Study

**DOI:** 10.3389/fpsyg.2020.589461

**Published:** 2020-12-08

**Authors:** Girija Kaimal, Katrina Carroll-Haskins, Arun Ramakrishnan, Susan Magsamen, Asli Arslanbek, Joanna Herres

**Affiliations:** ^1^Department of Creative Arts Therapies, College of Nursing and Health Professions, Drexel University, Philadelphia, PA, United States; ^2^International Arts + Mind Lab, Brain Science Institute, School of Medicine, Johns Hopkins University, Baltimore, MD, United States; ^3^Department of Psychology, The College of New Jersey, Ewing Township, NJ, United States

**Keywords:** virtual reality, drawing, fragrance, mixed method, well-being, affect, self-efficacy, stress

## Abstract

**Aims:**

In this pilot mixed-methods study, we examined the participants experiences of engaging in virtual drawing tasks and the impact of an olfactory stimulus (calming fragrance blend) on outcomes of affect, stress, self-efficacy, anxiety, creative agency, and well-being (satisfaction with life).

**Methods:**

This study used a parallel mixed-methods, simple block randomization design. The study participants included 24 healthy adults aged 18 to 54 years, including 18 women and six men. The participants completed two 1-h immersive virtual art making sessions and were randomly assigned to receive either a fragrance or a non-fragrance condition for the first session. Quantitative (standardized self-report measures) and qualitative (open-ended survey responses and virtual artwork) datasets were collected concurrently and integrated during data analysis.

**Results:**

The quantitative results indicated that the fragrance condition demonstrated a significant reduction in negative affect (e.g., feeling hostile, jittery, upset, distressed, etc.), namely, reduced feelings of negativity when compared to the non-fragrance condition. A trend toward improvement in self-efficacy was also seen in the fragrance condition. No significant changes were found for fragrance or non-fragrance conditions for positive affect, anxiety, and creative agency. The qualitative findings included five themes related to art making experiences in virtual reality in both conditions: fun and joy; novelty of virtual media, experimentation, and play; relaxation and calm; learning curve; and physical discomfort and disorientation. Four themes were identified for virtual art content and visual qualities: nature imagery, references to memories and personal symbols, fantasy and play within imagery, and depiction of everyday objects.

**Conclusions:**

Overall, the participants reported positive responses to the novel virtual art making experiences which were further heightened by the inclusion of the fragrance stimulus for negative affect. These preliminary findings need to be replicated with larger sample sizes to confirm the outcomes and the trends that were seen in this pilot study. Further research is recommended to examine the differences between experiences of virtual and traditional art media and to examine different olfactory stimuli promoting focus and concentration.

## Introduction

### Use of VR in Healthcare, Health, and Well-Being

Virtual reality (VR) technology is increasingly being used in healthcare settings. Virtual reality provides an experience that cannot be created in real life (Freeman et al., 2017). Mental health researchers have studied the applications of VR for the assessment, understanding, and treatment of physical and mental health disorders (Freeman et al., 2017; Felnhofer et al., 2019). Studies have shown that VR can be a useful tool in exposure therapy for phobias, including acrophobia (Emmelcamp et al., 2002), animal phobias (Morina et al., 2015; Suso-Ribera et al., 2019; Tardif et al., 2019), social anxiety (Felnhofer et al., 2019), and distraction from pain (Mosso Vazquez et al., 2019). Moreover, virtual environments have been created for treating post-traumatic stress disorder (Rizzo et al., 2014; Botella et al., 2015; Freeman et al., 2017; Weir, 2018). Other studies have incorporated VR for pain reduction with patients going through treatments such as chemotherapy (Chirico et al., 2020).

Virtual reality has also been used for mood induction in several studies (Baños et al., 2006; Herrero et al., 2014). Baños et al. (2006) developed a virtual environment (park with trees, water, and flowers) designed to induce mood (sadness, happiness, anxiety, and relaxation). The participants completed two 30-min “walks” through the virtual environment guided by a female voice according to the programmed emotional condition (Baños et al., 2006, p. 10). The results suggested that virtual mood induction procedures are effective in inducing both a target mood (sadness) and changing the induced mood to the opposite emotion (happiness) (Baños et al., 2006, p. 12). Herrero et al. (2014) investigated the efficacy of VR in promoting positive emotions with participants with fibromyalgia. A virtual environment (beach) was designed to induce positive emotion including projected videos, images, and audio. The findings demonstrated increased positive emotions, motivation, and self-efficacy (Herrero et al., 2014). Similarly, a quasi-experimental study by Shah et al. (2015) examined the feasibility and the effects of a VR-based stress management program for participants diagnosed with mood disorders in an inpatient hospital setting. This VR-based stress management program consisted of viewing three videos combining psychoeducation and relaxation techniques (abdominal breathing, muscle relaxation, and guided imagery) using a VR headset. The results demonstrated significantly lower depression and anxiety following virtual stress management sessions. Examining creativity and relaxation, Yang et al. (2018) conducted a study examining the effects of VR on undergraduate students. The experimental group participants were asked to complete an open-ended challenge to design wearable technology on a virtual mannequin using Google Tilt Brush, a virtual drawing software program. The control group participants created designs using paper and pencil. The findings suggested that the immersive three-dimensional experience promoted sustained attention and resulted in higher-quality creative products (Yang et al., 2018).

### Digital Artmaking and VR in Art Therapy

In line with the increased interest in incorporating technology into mental healthcare research, art therapists have also integrated digital media in their work (McLeod, 1999; Thong, 2007; Chilton et al., 2009). Art therapists have utilized computer technology in their clinical practices, including video editing and design programs such as Adobe Photoshop, Flying Colors, Paintbrush, Metacreations, and People Putty (McLeod, 1999; Thong, 2007). Most literature in digital art therapy refers to the use of two-dimensional digital screens, tablets, and stylus tools. In the use of such digital devices, the art making experiences occur in the context of pixelated visual works, not tactile as in traditional art making. Several articles and survey studies suggest that art therapists were initially cautious about welcoming technological media into the art therapy studio (Peterson et al., 2005; Asawa, 2009; Peterson, 2010), with concerns regarding the loss of tactile and sensual qualities in digital art making environments (McNiff, 1999). Nevertheless, the need for technology training for art therapy students was acknowledged by students (Orr, 2006), and views on virtual technologies have been changing, highlighting the importance of “broadening definitions of art materials and contexts” (Kapitan, 2007, p. 51). In the current climate of COVID-19, the use of technology in art therapy has been recognized, drawing attention also to ethical and safety concerns in the operation of these media (Alders et al., 2011).

Although the incorporation of digital technological tools has been used by art therapists for some years, there is limited and emergent literature discussing the use of VR in art therapy. Virtual reality is a relatively new medium that is different in that it allows for digital art making in three dimensions while also immersing the participant in a new digital visual environment (different from physical visual environment) (Hacmun et al., 2018; Kaimal et al., 2019). In using VR, individuals need to use a headset that transports them into a new environment that is imaginary/simulated visual and different from the physical environment that is retained in traditional art making. Therapeutically, VR can be used for receptive experiences like reflection and meditation in a new immersive environment or for creative self-expression using VR-specific painting and sculpting tools. Hacmun et al. (2018) highlight that VR can promote a sense of presence and allows for transformations of the sense of self. Despite the absence of physical art materials, the embodied environment that VR offers can enhance the participant’s presence within the artwork. According to Hacmun et al. (2018), this leads to an augmented experience of artistic creation that other artistic media cannot offer. Virtual reality mimics visual and motor signals, promoting an enhanced illusionary experience that makes the participant feel as if he/she is there (Riva et al., 2016). Lohrius and Malchiodi (2018) draw attention to the intuitive, accessible, and embodied aspects of art making in VR. Artmaking in VR can connect movement with visual art making by allowing the participants to incorporate their physical body to draw in fluid motions. Physical connection with the artistic creation in VR increases the participant’s engagement with the art making process (Lohrius and Malchiodi, 2018).

### Olfactory Stimulus in Virtual Reality

Engaging in VR involves detachment from real-life physical sensory experiences and shifts perception into digital visual and auditory stimuli (Hacmun et al., 2018; Kaimal et al., 2019). Olfactory stimuli can be a way to connect back to the senses when other senses like that of sight and spatial perception are altered in VR. Olfactory perception is complex, and although the mechanisms of signal transduction and mapping of odors are understood, the impact and the categorization of odors have been more challenging in comparison to visual and auditory senses, which can be classified by colors and decibels, respectively. Olfactory stimulation connects to the emotion areas of the brain such as amygdala, hippocampus, and orbitofrontal cortex (Haberly, 2001; Wilson, 2003). According to Krusemark et al. (2013), olfactory processing is dependent on emotional state and can be exploited by emotion induction. For instance, following an anxiety induction, natural odors can smell unpleasant and could be harder to detect. In a systematic review, Herz (2009) presents 18 articles that show that there is evidence demonstrating the impact of odors on mood, behavior, and physiology.

Studies have examined olfactory stimuli on outcomes of self-efficacy and mood. Baron (1990) assessed the impacts of artificial fragrances on self-efficacy and work performance. The participants were randomly assigned to either a pleasant or a neutral scent condition. The neutral scents (such as sesame oil, WD-40, wood workers glue) were pre-tested and categorized as being perceived as relatively neutral affective reactions (Baron, 1990). The participants exposed to pleasant scents (perfumes) were found to set higher goals on an assigned coding task and were more likely to adopt a more efficient strategy compared to the participants exposed to neutral scents. Moss et al. (2003) investigated the olfactory impact of lavender and rosemary essential oils on mood and cognition. Following the completion of cognitive assessments, the participants exposed to rosemary odor demonstrated increased alertness, whereas the participants in non-fragrance control and lavender conditions demonstrated decreased alertness. The participants exposed to either lavender or rosemary odors were found to be more content post-intervention as compared to the control condition. A randomized control trial examining the effects of lavender and lemon odors pre- and post- exposure to a stressor (cold pressor) found that exposure to lemon odor resulted in a significant positive mood when compared to lavender and a non-fragrance condition (Kiecolt-Glaser et al., 2008). In a randomized control crossover study investigating the impacts of yuzu (a Japanese floral citrus fruit) on outcomes of mood and stress, a significant decrease in mood disturbance and stress was found 30 min post-yuzu fragrance exposure (Matsumoto et al., 2014). The authors concluded that yuzu has a soothing effect and elicited a positive emotional response. Findings of a study examining positive and negative affect following exposure to six odors (citral, citronellol, phenylethyl alcohol, geraniol extra, vanillin, and nonalactone gamma) resulted in decreased positive affect and a significant moderate increase in negative affect specifically for phenylethyl alcohol odor (Gaby and Tepper, 2020). This extant olfactory research indicates that exposure to specific odors can influence affect.

Importantly, researchers have begun to examine the impacts of integrating olfactory stimuli with VR. To better understand human olfactory perception, Micaroni et al. (2019) designed an experiment using VR combined with an olfactory display (OD), a computed controlled device that releases fragrance. The authors recommended that future studies apply the OD prototype developed to disperse fragrance *via* air pump in synchronization with participant breath and virtual imagery *via* a head-mounted VR device (Oculus Rift). In a quasi-experimental trial by Cheng et al. (2020), VR was combined with aromatherapy for elderly participants in a long-term care facility. Aromatherapy was combined with synchronized virtual nature imagery, such as dispersing orange fragrance while the participants picked oranges in VR. The experimental group engaged in weekly 2-h VR aromatherapy sessions for a total of 9 weeks. The results demonstrated significant improvements in life satisfaction, happiness, sleep quality, and decreased stress.

As seen from the literature, further research examining the impact of creative expression in VR and olfactory stimuli to create immersive virtual experiences is needed especially as this relates to art therapy practice. In addition, arts-based intervention research needs to be able to be conducted such that the evidence can be applicable for clinicians. Impact thinking (developed at the International Arts + Mind Lab at Johns Hopkins University) is one such framework that was created in response to the increased awareness of and capacity for arts-based therapy in medicine (Magsamen, 2017). It is a consensus framework that applies rigorous brain science research methods to arts, architecture, and music interventions by engaging a broad and multidisciplinary team. Beginning with a problem identification workshop and collaborative discovery process and concluding with dissemination and scaling, impact thinking is designed to build open-source capacity and expertise and a research-to-practice pipeline for arts + mind research focused on outcomes.

In our study, we developed the experimental protocol in discussion with a team of interdisciplinary scholars and identified two drawing tasks with scripted directions and conditions that could be examined as systematically validated self-report measures which have previously been found to be effective in capturing changes as brief art making experiences (Kaimal and Ray, 2016; Kaimal et al., 2017). Drawing tasks and a fragrance stimulus were introduced to better understand what aspects of psychosocial functioning might be affected by the inclusion of olfactory stimuli. Given that the study set required a heavy VR headset and the challenge of an unfamiliar experience of art making in a novel context, we chose a relaxation-inducing scent in order to support and not cause additional stress for the participants.

We tested two main hypotheses regarding the effects of visual self-expression and fragrance on psychosocial functioning. Our first hypothesis was that the participants would experience improvements in psychological variables, including perceived stress, affect, general self-efficacy, and creative agency, and increases in life satisfaction. Our second hypothesis was that improvements in psychological variables would be larger when fragrance was added to the creative art sessions. Qualitative findings in the form of recurring themes in the artwork and narrative responses were expected to further explain the qualitative results and offer insights into the participants’ experiences.

## Materials and Methods

### Study Design

This study used a parallel mixed-methods, simple randomization block design to assess differences in perception of VR drawing tasks and an olfactory stimulus on outcomes of stress, affect, self-efficacy, anxiety, creative agency, and well-being (satisfaction with life). Qualitative (open-ended survey responses) and quantitative (self-report measures) datasets were collected concurrently and integrated during data analysis. This research is part of a larger study examining the outcomes of virtual drawing tasks on reward perception as measured using functional near-infrared spectroscopy (fNIRS). This paper will focus solely on self-report data of participant experiences engaging in virtual drawing tasks. The investigators received Institutional Review Board approval for this study.

### Setting

**FIGURE 1:**
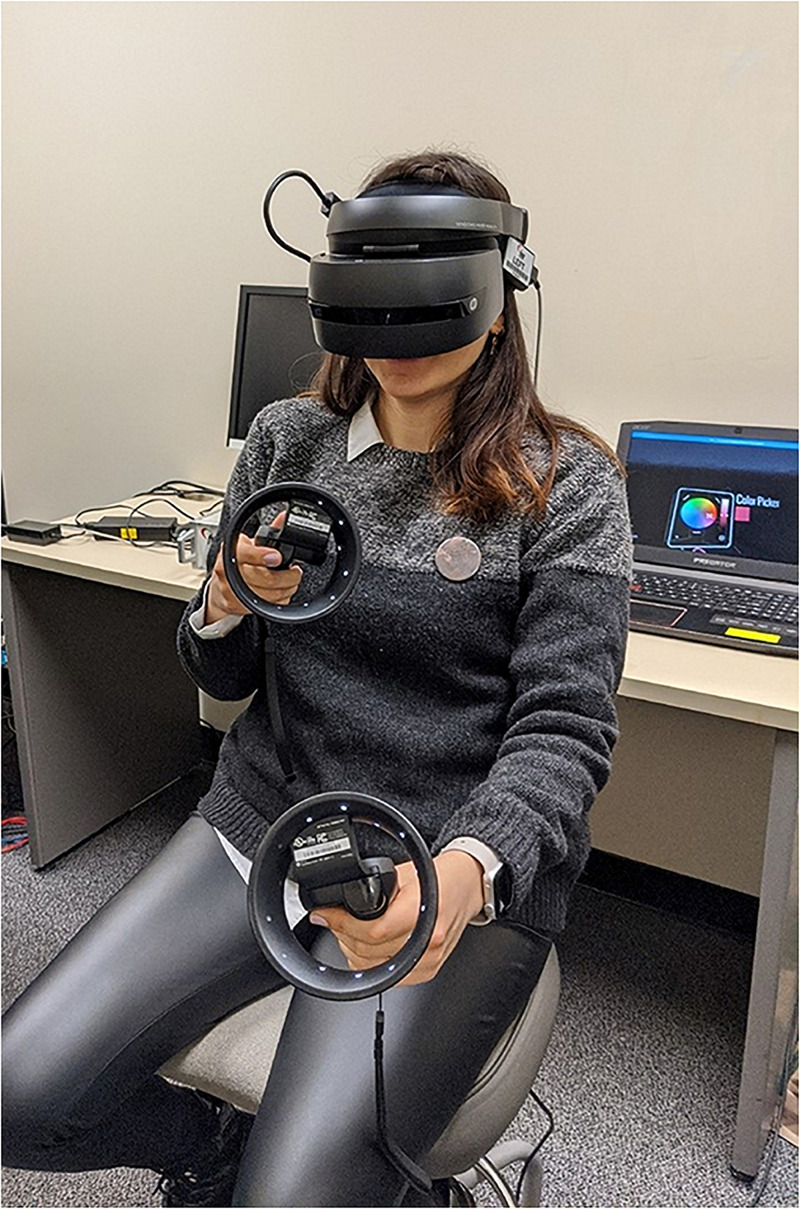
Windows mixed-reality headset and controls in lab setting.

All study sessions were facilitated in a dedicated lab room at a large urban university. The lab space contained VR hardware including the Windows Mixed-Reality headset and remote control devices running on a personal laptop with the following capabilities: (a) laptop PC: Acer Predator Helios 300 (G3-571-77QK), 15.6″ Full HD IPS, Intel i7 CPU, 16 GB DDR4 RAM, 256 GB SSD, GeForce GTX 1060-6 GB, VR Ready, Windows 10 64-bit, (b) HMD: Lenovo Explorer (G0A20001WW) Mixed Reality Headset (Figure 1; for further information on computer specifications needed to run Windows Mixed-Reality, see^1^). Tilt Brush by Google^2^, a virtual drawing software program, was used to create immersive 3-D images in VR.

Fragrance was diffused into the lab using a standard diffuser system (Ascents^®^ diffuser system by Aeroscena^®^) containing Aeroscena^®^ scent gel in the fragrance Calm No. 34. This fragrance contains a blend of essential oils consisting primarily of lavender as well as orange, juniper berry, patchouli, and ylang-ylang (the percentages were unavailable as fragrance blend is propriety; for further information, see^3^). Fragrance was diffused into the lab on alternating weeks and dissipated within 30 min after turning off the diffuser.

### Participants

The recruitment procedures included publicly displayed flyers in community spaces and approved online research webpages. The inclusion criteria included being a healthy adult between 18 and 70 years old. No prior artistic experience or VR experience was required. The exclusion criteria included any known fragrance allergies, history of seizures or head trauma, or issues precluding the ability to wear a VR headset. Thirty-eight participants expressed interest in the study, but 13 did not participate primarily due to scheduling conflicts or were non-responsive to follow-up emails to reschedule. One participant left the study due to momentary physical discomfort (nausea) associated with the VR immersive environment. Overall, 24 adults participated in the study, including 18 women and six men. Three participants only completed one session due to scheduling conflicts. The participants ranged in age from 18 to 54 years old, with an average age of 27.5. The participants were identified as White, including regional/ethnic specific identity (*n* = 11), African American (*n* = 3), Asian (*n* = 6), and of mixed race (*n* = 4). The participants were scheduled for two 1-h sessions scheduled at least 1 week apart and received $10 compensation for each session.

### Data Collection

The study participants were scheduled for two 1-h sessions. The participants were assigned through a simple randomization plan (alternating odd and even number assignments) to receive either fragrance or non-fragrance condition for the first session. The participants were blinded to the condition. The second session was scheduled for at least 1 week following the first session. The participants completed informed consent forms, a demographic form, and a brief screening form during the first session. The participants completed a battery of self-report measures prior to and immediately following the VR session and were asked to respond to all self-report measures to reflect their feelings in the present moment. At the end of each session, the participants responded to two open-ended narrative questions: (1) What was it like to make the artwork? and (2) Can you share what you created and/or what it represents for you?

### Measures

#### Patient-Reported Outcomes Measurement Information System Anxiety Short Form

The four-item short form of the Patient-Reported Outcomes Measurement Information System (PROMIS) tool was used to measure anxiety. This scale asks the participants to rate their perceptions of how frequently they experienced items such as “I felt uneasy” on a five-point scale from “never” to “always.” The short-form PROMIS tool was calibrated against legacy measures, and it demonstrates strong internal reliability of Cronbach’s α = 0.93 (Pilkonis et al., 2011).

#### Perceived Stress Scale

The 10-item Perceived Stress Scale (PSS) (Cohen et al., 1983) was used to measure the participants’ perceptions of stress in relation to their ability to cope with stressors. In a sample of 1,236 adults, the PSS was found to have moderate to good convergent validity and good internal consistency reliability of Cronbach’s α = 0.84 (Taylor, 2015). Construct validity demonstrated a high correlation between the State-Trait Anxiety Inventory and the PSS.

#### Positive and Negative Affect Schedule

The 20-item Positive and Negative Affect Schedule (PANAS) (Watson et al., 1988) was used to measure affect. The participants marked a five-point scale ranging from “not at all” to “extremely” regarding positive and negative feelings. Positive feelings are referenced on the scale using words like feelings such as enthusiastic, alert, inspired, etc., while negative feelings are captured using words like nervous, jittery, distressed, etc. The responses to the positive and the negative words are added to generate an overall score for positive affect and negative affect. In a large sample of the general adult population (1,003 participants), the two subscales have shown good internal consistency reliability of Cronbach’s α = 0.89 for positive affect and Cronbach’s α = 0.85 for negative affect (Crawford and Henry, 2004).

#### General Self-Efficacy Scale

The 10-item General Self-Efficacy (GSE) (Schwarzer and Jerusalem, 1995) was used to assess the participants’ self-perception of their ability to cope with challenging situations. The participants rated their responses on a four-point scale ranging from “not at all” to “exactly true” for items such as “I can always manage to solve difficult problems if I try hard enough.” The GSE has shown high internal consistency reliability, with studies across multiple countries in diverse adult populations finding Cronbach’s α from 0.86 to 0.94 (Luszczynska et al., 2005).

#### Creative Agency Scale

Creative agency was measured using a set of five items selected from scales of creative agency and identity (Tierney and Farmer, 2002; Beghetto, 2006), including self-perceptions of being able to generate novel ideas, ability to solve problems creatively, being imaginative, coming up with good ideas, and having good ideas. In a study of healthy adults, the creative agency scale items were found to have high internal consistency reliability (Cronbach’s α of 0.90) (Kaimal et al., 2017).

#### Satisfaction With Life Scale

The five-item Satisfaction With Life Scale (SWLS) (Diener et al., 1985) was used to measure self-perception of life satisfaction. The participants marked a five-point scale ranging from “strongly disagree” to “strongly agree” for items such as “My life has a clear sense of purpose.” In a large sample of a general adult population (2,524 participants), the German version of the SWLS was found to have good internal consistency (Cronbach’s α = 0.92) and correlated positively with social support (*r* = 0.39, *p* < 0.001, two-tailed) and negatively with depression (*r* = –0.44, *p* < 0.001, two-tailed) (Glaesmer et al., 2011).

### Procedures

**FIGURE 2:**
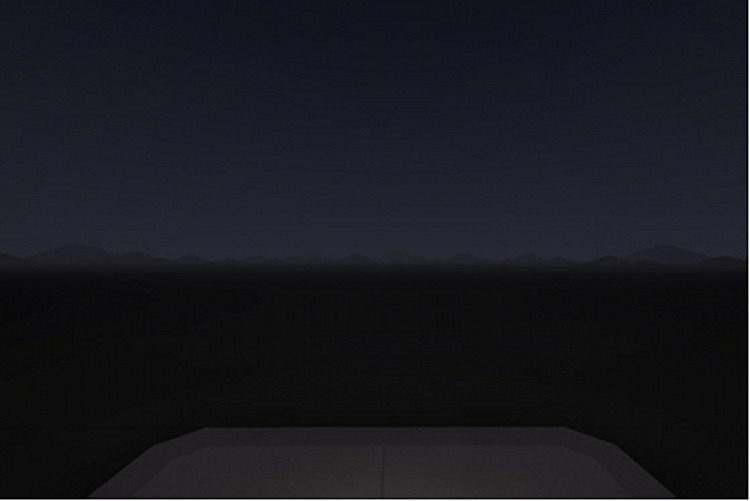
Tilt Brush default environment.

After completing informed consent and pre-survey measures, the participants engaged in a brief orientation with the facilitating art therapist to become familiarized with the VR headset and remote controls, art making features, and virtual environment within Tilt Brush (Figure 2). The participants remained seated for the entire session to reduce the risk of disorientation but were free to move their head and arms while drawing in VR. This was essential to orient and familiarize the participants with the study protocols that followed. The facilitator was an art therapist proficient in the technology and could guide the participants in the options and controls. Thereafter, the participants completed two drawing tasks in VR: (1) creating a spontaneous drawing based on an adaptation of the scribble drawing technique, an approach frequently used in art therapy, and (2) tracing basic shapes on a pre-drawn template. These were selected to provide two different visual expressive experiences that were similar in time duration. The tasks included scripted directives to ensure similarity of experience in completion of the tasks within the duration of the session. The participants completed both drawing tasks twice during each session. The Tilt Brush tool allows for a range of pen, brush, color, density, and textural options akin to digital drawing tools. In addition, there are animated options like stars, dots, etc., and sound effects that can add to the visual immersive art making experience. Once the session was completed, the participants were assisted in removing the headsets and offered a few minutes of re-orienting to the room prior to completing the post-surveys.

### Data Analysis

#### Quantitative Data Analysis

Paired-samples *t*-tests examined changes in outcome variables from pre- to post-VR drawing tasks in both olfactory conditions (when the tasks were performed with a fragrance vs. without). Repeated-measures analysis of variance (ANOVA) tested the interaction between the effects of the VR drawing tasks (pre–post change) and the olfactory conditions, controlling for task order. Significant interactions were graphed for interpretability. An *a priori* power analysis determined that the repeated-measures ANOVA had 82% power to detect a medium effect.

#### Qualitative Data Analysis

Thematic analysis (Braun and Clarke, 2012) was used to code the narrative data and virtual artwork through the following phases of analysis: (1) familiarizing with the data, (2) generating initial codes, (3) searching for themes, (4) reviewing potential themes, (5) defining and naming themes, and (6) completing the final report. Open-ended narrative responses were coded for experiences of virtual art making as described by the participants. Virtual art data were coded for both visual qualities and content of the images. The research team independently developed initial codes for both narrative responses and virtual artwork and then compared the results to ensure credibility (Lincoln and Guba, 1985). Codes were then compiled into a matrix to identify recurrent themes for both fragrance and non-fragrance conditions. Themes were reviewed, and consensus was reached by the research team.

#### Integration of Qualitative and Quantitative Data

The study used a parallel mixed-methods design (Creswell and Clark, 2011). Qualitative and quantitative data were examined together to better understand the findings. The qualitative responses helped identify the preferred themes of the participants, confirmed some of the quantitative results, and highlighted the unique aspects of VR art making that warranted further research.

## Results

### Quantitative Results

**FIGURE 3:**
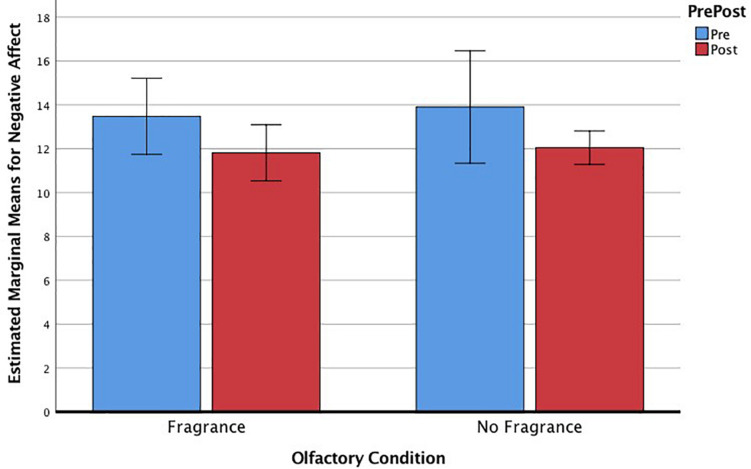
Estimated marginal means, controlling for task order, for negative affect across repeated-measures assessments, and olfactory conditions.

Paired-samples *t*-tests examined the main effects of the VR drawing tasks on outcomes of anxiety, stress, affect, self-efficacy, creative agency, and satisfaction with life. The results of the paired *t*-test (listed in Table 1) showed that there were significant changes following the VR drawing tasks in the expected directions, except that there was no change in positive or negative affect in the non-fragrance condition and no change in creative agency in either condition. Although the paired *t*-tests suggested differences between the fragrance and the non-fragrance conditions, we needed to test whether the differences were statistically significant *via* interactions between the within-subject effects of the creative arts session and the between-subject effects of fragrance. A series of repeated-measures factorial ANOVAs, controlling for task order, showed that the main effects of the VR drawing tasks on anxiety [*F*(1,19) = 0.71, *p* = 0.410], stress [*F*(1,19) = 0.75, *p* = 0.399], positive affect [*F*(1,19) = 0.059, *p* = 0.811], self-efficacy [*F*(1,19) = 2.49, *p* = 0.131], creative agency [(*F*(1,19) = 2.13, *p* = 0.161], and satisfaction with life [*F*(1,19) = 0.57, *p* = 0.458] did not significantly differ across the olfactory conditions (when the tasks were performed with a fragrance vs. without). However, a significant interaction effect for negative affect [*F*(1,19) = 6.99, *p* = 0.016, η_*p*_^2^ = 0.27] showed that, while the participants experienced reductions in negative affect in the fragrance condition, they did not experience significant reductions in negative affect in the non-fragrance condition (Figure 3).

**TABLE 1:** Results of paired-samples *t*-test showing the mean differences (*M*_*diff*_) in psychological outcomes.

**Variable**	***M*_*pre*_**	**SD_*pre*_**	***M*_*post*_**	**SD_*post*_**	***M*_*diff*_**	**SD_*diff*_**	***t*(22)**	***t*(21)**	***p***
**Fragrance condition**									
Anxiety	6.74	2.88	5.52	1.93	1.22	2.30	2.54		0.019
Perceived stress	12.52	7.20	9.87	7.39	2.65	4.90	2.60		0.016
Positive affect	31.74	8.72	34.61	9.98	−2.87	5.68	−2.43		0.024
Negative affect	14.22	4.52	12.00	2.86	2.22	3.12	3.41		0.003
General self-efficacy	32.48	6.40	33.61	5.99	−1.13	2.56	−2.12		0.046
Creative agency	20.65	3.49	20.48	3.98	0.17	1.40	0.59		0.558
Satisfaction with life	26.91	5.69	28.35	5.45	−1.44	1.88	−3.66		0.001
**Non-fragrance condition**									
Anxiety	6.45	2.91	5.41	1.84	1.05	2.61		1.88	0.074
Perceived stress	12.05	7.69	8.73	6.02	3.32	6.01		2.59	0.017
Positive affect	32.59	7.90	33.41	10.39	−0.82	8.34		−0.46	0.650
Negative affect	13.73	5.80	12.05	1.70	1.68	5.46		1.44	0.164
General self-efficacy	32.32	7.52	34.23	6.58	−1.91	3.50		−2.56	0.018
Creative agency	20.41	3.78	21.55	3.50	−1.14	2.678		−1.99	0.060
Satisfaction with life	26.82	5.71	28.73	5.69	−1.91	3.80		−2.35	0.028

### Qualitative Findings

A thematic analysis revealed themes common to both conditions (fragrance and non-fragrance) related to virtual art making experiences and art content. An analysis of the narrative responses indicated the following five themes relating to experiences of virtual art making: fun and joy; novelty of virtual media, experimentation, and play; relaxation and calm; learning curve; and physical discomfort and disorientation. An analysis of the content and visual qualities within the virtual art data revealed four themes in the content: nature imagery, references to memories and personal symbols, fantasy and play within imagery, and depiction of everyday objects.

#### Themes Relating to Virtual Artmaking Experiences

Most participants (*n* = 22) described virtual art making as fun and enjoyable:

“It was very interesting and fun. I really enjoyed painting in 3D. Definitely a different art experience than anything I have ever done before.”

—Non-fragrance condition

The participants (*n* = 20) frequently expressed a sense of play and experimentation due to the novelty of virtual media, specifically the ability to create 3-D artwork and explore the variety of different tools offered within Tilt Brush:

“I liked being able to make 3D things w/o all of the set-up that’s involved in making physical art. It felt really freeing to be able to just erase and start over and also to explore all of the brush types.”

—Fragrance condition

Some participants (*n* = 5) described feeling calm and relaxed while engaged in virtual art making:

“Today, I felt more relaxed. I slowed down and was more comfortable. I definitely focused on the art making and not distracted by anything else.”

—Fragrance condition

“Making the artwork releases my stress, gets my mind out of my daily stresses”

—Fragrance condition

A learning curve was experienced by some participants (*n* = 5), including challenges in adapting to the virtual hardware (remotes and headset) and the software (tools and art making processes within Tilt Brush). The novelty of the media means that some time and teaching are needed by the facilitating art therapist before the participant can fully engage with the experience, including having adequate time to learn and create.

“It took a few minutes to get comfortable with the VR tools. I have never tried it before, so it was new and different.”

—Non-fragrance condition.

“Sometimes it was a little frustrating because I felt like I couldn’t draw as precisely as I wanted to.”

—Fragrance condition.

The participants (*n* = 5) also expressed improved ability to engage with the tools over time after earlier drawing tasks and during the second session:

“This time I was more comfortable and confident in the tools. I had a clear sense of what I wanted to do and could accomplish it.”

—Fragrance condition.

“Having experienced VR before, I came in with an idea of what to do today, and it felt rewarding that I could draw what I wanted.” —Non-fragrance condition.

“In the second trial, I had a better idea in using the remote controls”

—Non-fragrance condition.

Some participants (*n* = 5) described experiences of physical discomfort and disorientation. The participants described experiences of physical discomfort in relation to the heaviness of the VR headset as well as experiencing pressure due to wearing a combination of the fNIRS headband and VR headset.

“[By the end of the session] my head hurt so it was difficult to focus on the artwork.”

—Non-fragrance condition.

“I could do it for hours if the headset wasn’t that heavy.”

—Fragrance condition.

“When I was instructed to do a scribble, I made a large scribble around where I sat. It became exhausting to look at it. It was electric waves.”

—Fragrance condition.

#### Themes Relating to Artwork Content

**FIGURE 4:**
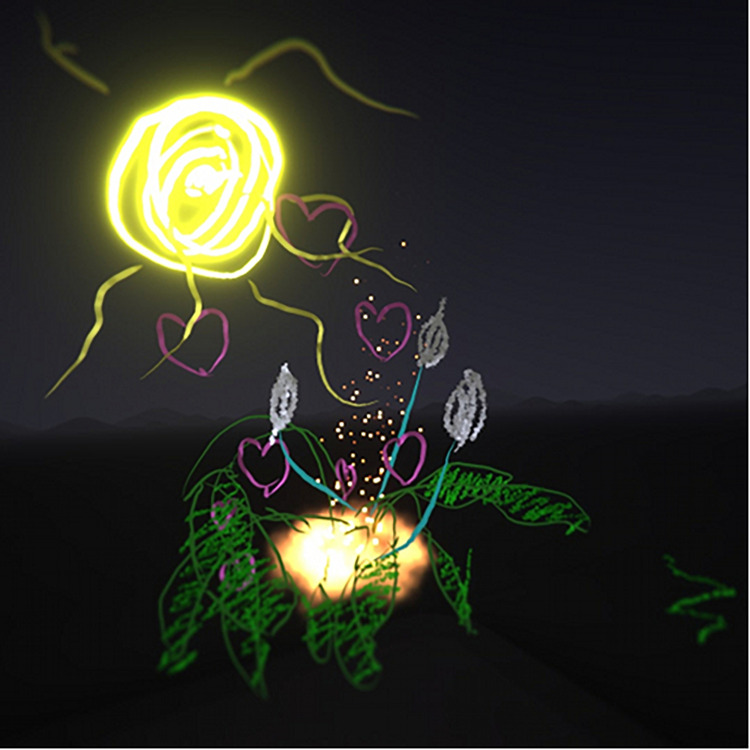
Nature image with sun, flowers, and grass.

**FIGURE 5:**
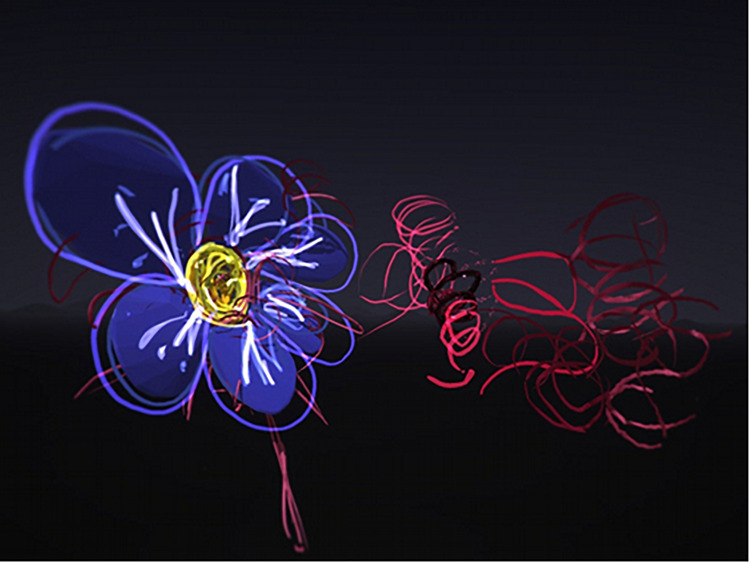
Flower drawing.

Several participants (*n* = 17) included nature themes within the virtual imagery. Flowers were commonly drawn by the participants (Figures 4, 5). Other nature images included landscapes with mountains, aquatic scenes including fish and boats, rainbows, forests, and animal figures.

**FIGURE 6:**
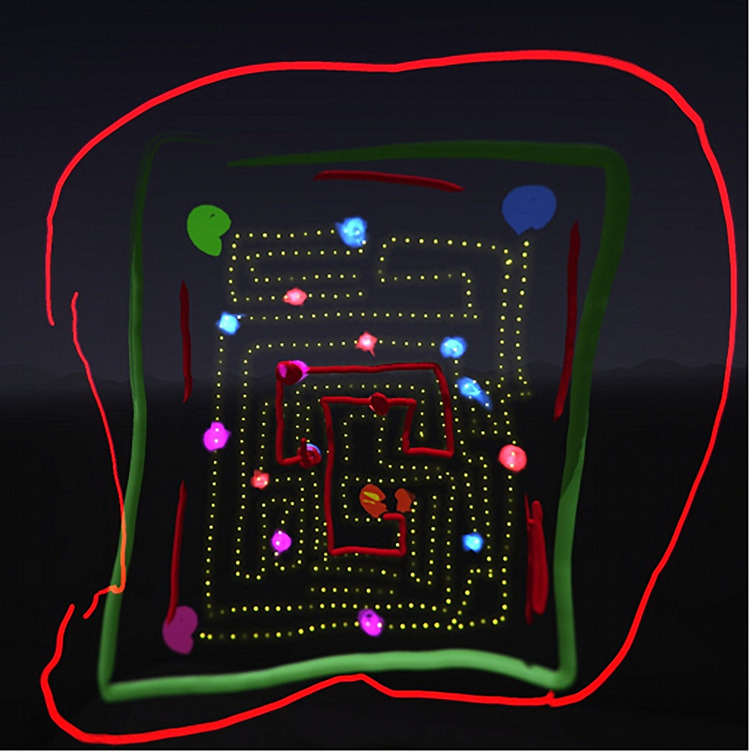
Drawing of a videogame from a participant’s childhood (Pacman).

Most participants (*n* = 20) included content relating to memories and/or personal symbols in their artwork. These images related to childhood memories, including drawing in art styles reminiscent of one’s childhood. The participants created images related to memories of winter holidays, symbols of familial relationships, and media such as one participant’s drawing of a favorite videogame played in childhood (Figure 6). Personal symbols were created through self-portraits, objects represented favorite hobbies and personal interests (sports and TV shows), and images meant to represent goals and aspirations.

**FIGURE 7:**
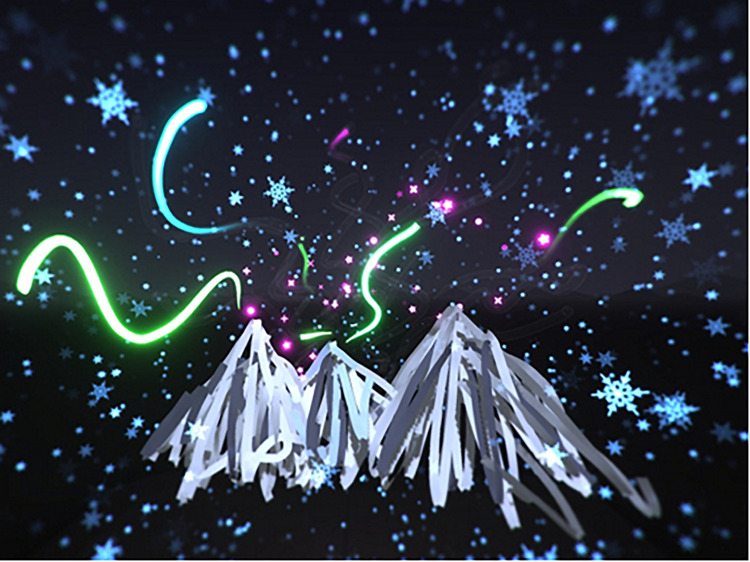
Fantasy depiction of the aurora borealis.

**FIGURE 8:**
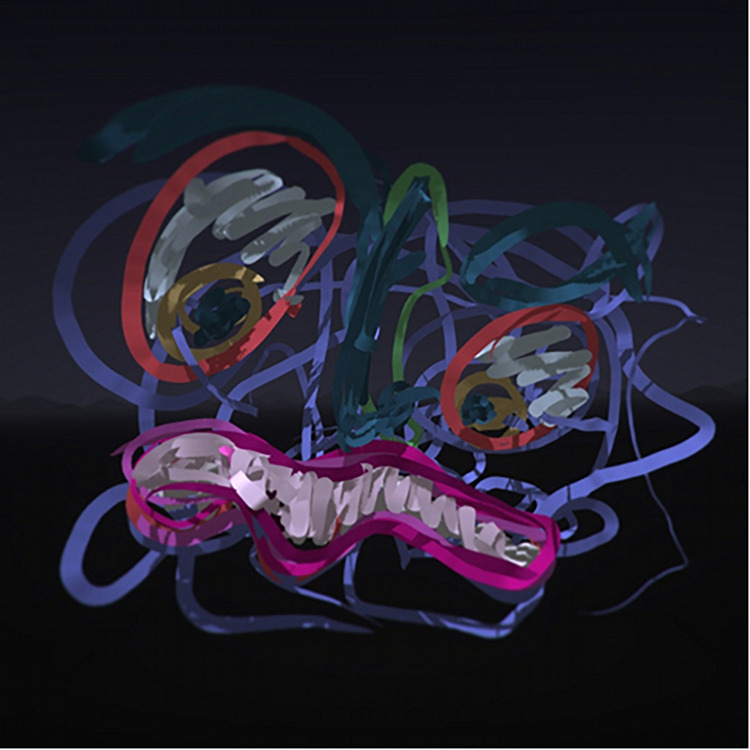
Depiction of a fantasy monster.

Artwork including aspects of fantasy, imagination, and play were created by the participants (*n* = 14). Fantasy imagery included vibrant drawings depicting fantasy creatures and scenes set in space, underwater environments, and the aurora borealis (Figure 7). The participants created playful and experimental images in which they tested various brushes and colors both spontaneously and intentionally. Some participants created spontaneous imagery while experimenting with brushes by finding various shapes and creating objects or creatures (Figure 8).

**FIGURE 9:**
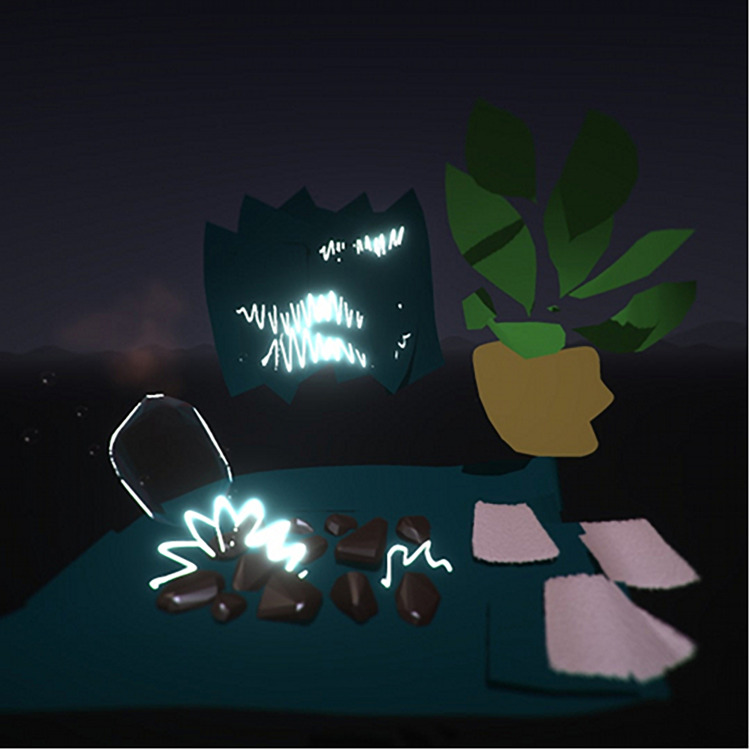
Desk with plants.

Artwork representing everyday objects were created by some participants (*n* = 7). Drawings of everyday objects included images of a pencil, food items, a car, a bus, a participant’s desk at work, and plants (Figure 9). The participants described these everyday objects as items that they used frequently or items representing work or hobbies.

As can be seen from the qualitative findings, the participants reported enjoying the creative visual expression activities, confirming at least partly that negative affect was reduced. A few participants reported discomfort with the VR headset, but the trends in improvement in self-efficacy were also seen mirrored in the references to the learning curve and novelty of the experience. Overall, visual imagery themes were seen in both conditions, indicating some similarities in both the fragrance and the non-fragrance conditions. Some themes like fun and joy, novelty, and fantasy and play were, however, referenced a few more times in the fragrance condition (Table 2).

**TABLE 2:** Presence of qualitative themes by condition.

**Qualitative themes**	**Fragrance condition (*n* = 23)**	**Non-fragrance condition (*n* = 22)**
Fun and joy	19	15
Novelty of virtual media, experimentation, and play	18	14
Relaxation and calm	4	2
Learning curve	3	3
Physical discomfort and disorientation	4	3
Nature imagery	12	11
References to memories and personal symbols	16	15
Fantasy and play within imagery	11	7
Depiction of everyday objects	5	2

## Discussion

In this mixed-methods pilot study, we examined the participants’ experiences and outcomes related to visual art making in VR. We tested two hypotheses related to visual art making using VR: (1) that there would be positive changes in the participants” responses on standardized measures of affect, stress, self-efficacy, anxiety, and creative agency and (2) that these changes would be enhanced with a fragrance stimulus. For the first hypothesis, significant changes were seen following the VR drawing tasks for all variables, except for affect in the non-fragrance condition, and there was no change in creative agency in either condition. For hypothesis 2, which examined changes between conditions, only negative affect was found to be significantly improved in the fragrance condition. This indicates that the experience of art making in VR was beneficial and that the addition of fragrance led to a significant change for reductions in the negative affect. A trend was also seen in improvements in self-efficacy in the fragrance condition, indicating that the participants felt a greater sense of self-confidence, but this requires further testing to be confirmed. Interestingly, there were no significant changes in positive affect, anxiety, and creative agency. This might have been because of the short span of art making session as well as the fact that we did not capture differences in the participants experiences between the drawing tasks.

In qualitative responses, the participants reported enjoying the scripted spontaneous self-expression task more than the rote tracing task. The spontaneous virtual drawing task was found to invite more open-ended expression. The participants reported a sense of playfulness and experimentation for the spontaneous virtual drawing task in contrast to the rote tracing task, which was found to be repetitive and restrictive by several participants. Additionally, many participants expressed a desire for more time to engage with the virtual art making tools in Tilt Brush during the spontaneous self-expression task. Overall, the participants described a sense of increased confidence in using the virtual tools to create imagery during the second session. In previous studies, we have participants reporting references on self-reflection (Hacmun et al., 2018; Kaimal et al., 2019) and unique perspectives, which was further confirmed in the creative expressive tasks in this study.

The VR artwork themes are also of note. Given that the background color of a night-like horizon was the starting screen, it is possible that this led to images of fantasy as depicted in many of the artworks. The VR medium likely lent itself to creating imaginal landscapes as well as memories. In previous studies where we used traditional art media (Kaimal and Ray, 2016; Kaimal et al., 2017), the participants tended to create imagery which had more refences to actual events or objects in everyday life. Creating in VR appears to have evoked more imagery and fantastical scenes and is an outcome that has potential educational and clinical applications for activating imagination in the participants. In addition, VR might have resulted in an arousal response somewhat dissimilar to working in traditional or digital art media. This might explain how the reduction in stress was not as significant as seen also in Yang et al. (2018). Very few participants in the study referred to prior experiences with art making. In previous studies, we have often found that the participants often referenced fears and inhibitions around a self-perceived lack of artistic skills (Kaimal and Ray, 2016; Kaimal et al., 2017). This was not seen much in our studies (Kaimal et al., 2019), including the current, perhaps because VR is not typically associated with artistic expression. This might offer an opportunity for involving participants in creative self-expression without the burden of self-criticism and expectation of skills that they might place on themselves with traditional media.

The participants were blinded to the fragrance stimulus itself in that they did not know in which of the two conditions they might encounter the fragrance stimulus. It was diffused in the room, and not all the participants noticed the fragrance. This makes the findings on the impact of fragrance particularly salient and minimizes bias in self-reports on the impact of this stimulus. The results might have been different if the fragrance was placed more closely to the participants (e.g., in a pendant-type option) such that they could access it more readily or if they were offered different ways to engage with the fragrance stimulus, including voluntary access to the fragrance.

### Implications and Recommendations for Further Research

This was a pilot study, and the findings need to be replicated with larger sample sizes to confirm the results and further test outcomes where trends were seen. In addition, differences between the drawing tasks need to be examined more closely to better understand the experiences. Examining the differences in the participants’ responses between the two tasks might have indicated more distinct trends, including the impact of fragrance based on tasks. The participants reported mostly all positive responses to the VR art making experience, but this might have been due to the novelty of the experience compared with traditional art media. Further research might examine how these differences play out when compared with traditional art media. Additional examination of measures related to well-being and novelty as well as experiences of imagination can be included in future studies. Measures that capture momentary changes in mood and mental states might also offer more precise outcomes compared with some of the more global outcomes seen in this study. Some participants reported finding the sessions too short, and the outcomes might have varied again if the participants felt that they had enough time to complete their artworks. The novelty of the VR media also means that the participants need time to familiarize themselves and get comfortable with this new drawing and expressive tool. The facilitating art therapist should be proficient in the technology in order to facilitate the patients’ experience. Fragrance, especially in the mild, natural, and diffused form, seems to be beneficial to the participants. Given that the VR environment can be disorienting for some individuals, it is advised that the experience be initiated in a seated position and then moved to an active standing posture as needed. The facilitating art therapist needs to ensure the overall comfort and safety of the participant, including providing enough physical space so that the individual in the VR headset does not hurt himself by bumping into objects in the room. With regards to the fragrance stimulus, this study used a specific product aimed at promoting calmness, and fragrance was diffused in the room. This indicates that a mild, diffused fragrance can enhance the participants’ experiences. Future studies might examine differences with other olfactory stimuli directed more at promoting focus and concentration. The medium of art making in VR is fundamentally different than working with traditional art media, and further examination of a taxonomy of themes and artwork content, especially the fantastical and the imaginative components seen in the VR artwork, is worthy of further inquiry. Overall, this research study used the impact thinking approach in order to support the translational scientific process, making it inclusive, relevant, and actionable. With this approach, we aim to move beyond studies that begin and end in a lab to solve real-world, urgent problems and pave a path for broad implementation. Specifically, we initiated the process of impact thinking with this study such that ongoing iterative examination of arts-based approaches could be applied to establish innovative evidence-based clinical applications. To date, we are not aware of any studies that have systematically examined how art therapy can be integrated into a VR-based expression to enhance patient care. This approach and the initial findings could help expand arts therapy opportunities to clinical populations, including those in physical rehabilitation and those facing psychological stressors and challenges. Given some of the creative opportunities inherent in VR and sensory stimuli, impact thinking can help expand the applications.

### Limitations

The study has many limitations, including a small sample size comprised of mainly college-educated female-identified participants that limit the generalizability of the results. One participant left the study due to cybersickness (dizziness and nausea) during and immediately following the VR drawing tasks. This discomfort was only monetary, and at follow up, the participant reported feeling fine. Studies have indicated that cybersickness occurs with low frequency (Shah et al., 2015; Chirico et al., 2020). Some participants reported experiencing headaches and overheating during the VR sessions. Cybersickness was likely mitigated for many participants through remaining seated while in VR and taking breaks (less than 5 min) between virtual drawing tasks. These hardware- and media-related experiences are to be considered in future studies. A limitation is also that, even though there were two drawing tasks, we did not collect survey feedback data for each drawing task. That is something we expect to do in the future with different measurement tools.

Some questions on the self-report measures were found to be not suited for some participants with English as a second language (ESL). All self-report measures were provided in English. Some ESL participants required assistance from the facilitators to translate or clarify some words and statements on self-report measures. Another limitation is the use of one fragrance blend, consisting primarily of lavender, developed to induce a calming response. Inclusion of different odors such as citrus scents (orange, lemon, and yuzu) and other fragrance blends may have yielded different results (Baron, 1990; Kiecolt-Glaser et al., 2008; Matsumoto et al., 2014; Cheng et al., 2020).

The qualitative results indicated that several participants drew nature-related images. The participants may have been primed during the drawing tasks due to the default environment containing a horizon line with gray objects appearing to be hills or mountains within Tilt Brush. Also, many images contained references to winter holidays and seasonal weather, which may have been primed due to the timing of data collection during fall and winter seasons. Time constraints (5 min per drawing task) may have impacted the drawing content. The participants may have created more detailed drawings with additional time. Overall, clinicians who would like to use VR with patients and clients need to be proficient in the media themselves and be prepared to teach patients how to use it effectively (such that it does not cause excessive disorientation or physical discomfort). In using fragrance stimulus in the rooms, ensure that it is from natural sources and also is not something that has been known to generate any adverse reaction.

## Conclusion

In this mixed-methods pilot study, we examined outcomes of visual self-expression using VR tools, with and without fragrance stimuli. The findings indicate that the participants reported reductions in negative affect and trends toward improvements in self-efficacy in the fragrance condition compared with the non-fragrance condition. The participants also preferred making creative works compared to tracing tasks. Artwork created in VR tended to include themes of nature, fantasy, memories, and everyday objects. Overall, the participants reported enjoying the novel experience, and a small number reported physical discomfort with the physical apparatus of the VR headsets. The participants do need to have enough time to familiarize themselves, and the art therapist should be proficient in the technology in order to facilitate the patient/client experience. Fragrance, especially in the mild, natural, and diffused form, seems to be beneficial to the participants. Further research with larger samples and more sensitive measures is needed to confirm these preliminary findings.

## Data Availability Statement

The data can be made available with written requests to the first author.

## Ethics Statement

The studies involving human participants were reviewed and approved by the Drexel University Institutional Review Board (IRB). The participants provided their written informed consent to participate in this study. Written informed consent was obtained from the individual(s) for the publication of any potentially identifiable images or data included in this article.

## Author Contributions

GK, AR, and SM contributed to the study conceptualization and design. KC-H led the data collection and consent process, and AA supported both. GK, KC-H, and AA conducted qualitative data analysis. JH performed the statistical analysis. GK, KC-H, JH, and AA wrote sections of the manuscript. All the authors contributed to manuscript revision and read and approved the submitted version.

## Conflict of Interest

The authors declare that the research was conducted in the absence of any commercial or financial relationships that could be construed as a potential conflict of interest. The reviewer CS declared a past co-authorship with one of the authors GK to the handling editor.

## Footnotes

1 https://www.microsoft.com/en-us/windows/windows-mixed-reality

2 http://www.tiltbrush.com

3 https://www.aeroscena.com/
